# Assessing the credibility of a drug’s effects: identification and judgment of uncertainty by the Dutch Medicines Evaluation Board

**DOI:** 10.3389/fmed.2024.1409259

**Published:** 2024-07-17

**Authors:** Joyce M. Hoek, Jonna Brenninkmeijer, Ymkje Anna de Vries, Rob R. Meijer, Don van Ravenzwaaij

**Affiliations:** ^1^Department of Psychology, University of Groningen, Groningen, Netherlands; ^2^Division of Pharmacoepidemiology and Clinical Pharmacology, Utrecht Institute for Pharmaceutical Sciences, Utrecht University, Utrecht, Netherlands; ^3^Department of Ethics, Law and Humanities, University of Amsterdam, Amsterdam UMC, Netherlands; ^4^Department of Child and Adolescent Psychiatry, University Medical Center Groningen, Groningen, Netherlands

**Keywords:** uncertainty, benefit–risk, regulatory decision making, credibility, regulatory authorities

## Abstract

Medicine regulators need to judge whether a drug’s favorable effects outweigh its unfavorable effects based on a dossier submitted by an applicant, such as a pharmaceutical company. Because scientific knowledge is inherently uncertain, regulators also need to judge the credibility of these effects by identifying and evaluating uncertainties. We performed an ethnographic study of assessment procedures at the Dutch Medicines Evaluation Board (MEB) and describe how regulators evaluate the credibility of an applicant’s claims about the benefits and risks of a drug in practice. Our analysis shows that regulators use an investigative approach, which illustrates the effort required to identify uncertainties. Moreover, we show that regulators’ expectations about the presentation, the design, and the results of studies can shape how they perceive a medicine’s dossier. We highlight the importance of regulatory experience and expertise in the identification and evaluation of uncertainties. In light of our observations, we provide two recommendations to reduce avoidable uncertainty: less reliance on evidence generated by the applicant; and better communication about, and enforcement of, regulatory frameworks toward drug developers.

## 1 Introduction

In most countries, medicinal products, in the EU defined as “any substance or combination of substances presented for treating or preventing disease in human beings” [Directive 2001/83/EC Art 1 (2)] (1) can only be marketed after evaluation by a regulatory authority, such as the European Medicines Agency (EMA) or a national competent authority (NCA). These authorities assess multiple aspects of a drug, including preclinical data from animal studies; manufacturing quality; pharmacokinetic and pharmacodynamic data; and efficacy and safety. In this regard, regulators perform a benefit–risk assessment to determine whether a drug can be granted marketing authorization, that is, whether the benefits outweigh the risks. The present article focuses on the assessment of efficacy and safety.

Regulatory authorities assess the benefits and risks of a drug based on a dossier submitted by the applicant who has conducted studies to support their claims about the effects of a drug. However, the evidence presented in a drug’s dossier necessarily comes with uncertainty (2), since uncertainties and scientific knowledge are inextricably linked to each other (3). Broadly speaking, uncertainty refers to what is unknown or unknowable, although many different definitions are used (4, 5). Regulators can face countless uncertainties, for instance due to unavailable, inaccurate or conflicting information or due to random variation. They may also face uncertainty about how a decision will play out in the future (4). Uncertainty poses a difficulty for medicine regulators who need to evaluate a drug’s benefit–risk balance. Regulators not only need to judge whether the favorable effects of a drug outweigh its unfavorable effects, they also need to judge the credibility of these claimed effects (2). This means that regulators need to identify uncertainties, assess their importance, and potentially propose mitigation strategies.

Both the evaluation of the balance of favorable and unfavorable effects and the evaluation of the credibility of these effects rely on judgment. It is therefore not surprising that different regulatory authorities do not always reach the same conclusions when reviewing the same drug, although they have a high concordance rate (6, 7). Different authorities may value aspects of the drug (e.g., its reported effects) and the drug’s context (e.g., unmet need) differently. Although many factors can affect a regulator’s decision making [e.g., see (7–9)], little is known about how regulators assess a drug’s evidence and uncertainties in practice.

### 1.1 Aim

This article therefore aims to answer the question: how do regulators assess the credibility of an applicant’s claims about the benefits and risks of a drug, and how do they identify uncertainties that may invalidate these claims? To answer these questions, we use material from an ethnographic study of assessment procedures we conducted at the Dutch national competent authority: the Medicines Evaluation Board (MEB). Based on our qualitative study of regulatory decision-making, we describe what makes a claimed effect credible to regulators; and in contrast, what makes regulators inclined to question a claim. We describe how regulators identify and evaluate uncertainty in practice. Balancing of different (un)favorable effects of a drug and uncertainty mitigation strategies are outside the scope of this research (although we touch upon these in the discussion).

Our analysis demonstrates that the identification and evaluation of uncertainty in medicine evaluation is context-dependent by illustrating the importance of regulatory experience and expertise. We thereby add to previous studies that have suggested that the acceptability of a specific level of uncertainty (10) and the relevance of uncertainties for the regulator’s decision-making (4) are context-specific. Thus, we provide more clarity on how and why the context of an assessment can influence the regulator’s dealing with uncertainty. In the remainder of this introduction, we briefly discuss the organization of medicine evaluation in Europe and how the Dutch MEB fits into this organization. We also briefly discuss prior theoretical work on the concept of uncertainty that has informed our analysis.

### 1.2 Medicine evaluation at the Dutch MEB

In Europe, medicines can enter the market via centralized, decentralized or national procedures. When a drug obtains marketing authorization via the central procedure, EMA’s Committee for Medicinal Products for Human Use (CHMP) is responsible for final assessment of the dossier submitted by the applicant (after which official approval is granted by the European Commission). Two CHMP representatives from each member state take part in the monthly CHMP meeting to discuss medicine assessments. A drug’s assessment typically takes place in three rounds, and it takes about a year to complete (11). For each medicine, two countries are appointed to prepare the detailed assessment of a drug (the rapporteur and co-rapporteur), while other countries are referred to as “concerned” member states. Although the procedure is centralized, NCAs have an important role in the procedure. When the Netherlands is appointed as one of the rapporteurs, the Dutch MEB prepares the assessment report. When the Netherlands is a concerned member state, the MEB also carries out an evaluation of the drug and of the rapporteur’s assessments prior to the CHMP discussion. In addition to the centralized procedure, a drug can gain approval in multiple (but not all) EU countries through either the decentralized or mutual recognition procedure. In this case, the NCA of one Reference Member State (RMS) conducts the primary assessment, and the marketing approval will also apply to one or several Concerned Member States (CMS) (12). Finally, the NCA of a particular country also carries out the assessment of drugs that will be marketed only in that country through a national procedure (13). In addition to assessing new drugs for marketing authorization, the EMA also considers variation or extension applications to drugs that have already been authorized (e.g., registration for a new indication) (14).

The Dutch MEB consists of two parts: the Agency consists of teams of assessors that prepare assessment reports, while the Board is responsible for the final recommendation regarding a drug. Each assessment involves one or multiple quality, non-clinical, pharmacokinetics, methodological, clinical and pharmacovigilance assessors. We focus on the work of the clinical assessors. When the Netherlands is RMS or (co-)rapporteur, the clinical assessors of the MEB write a report describing their findings and whether they consider the benefit–risk ratio of the drug to be positive or negative. In addition to a description of the data, and a judgment on the benefit–risk ratio, the assessment report also contains a list of questions (LoQ) the regulator wants the applicant to address during the next assessment round. The list of questions includes major objections (MOs)—serious problems that can prevent marketing authorization if not resolved—and other concerns (OCs)—less serious problems that should still be solved before a drug can be approved but that will not prevent a positive outcome. These assessment reports and the list of questions are then discussed during a Board meeting, which may result in modifications of the assessment. In the case of national and decentralized procedures in which the Netherlands acts as the RMS, the Board has the final say in the assessment. In centralized procedures, the Board issues an advice that will be discussed in the CHMP.

### 1.3 The concept of uncertainty

The term “uncertainty” is defined and used differently in different fields. Our understanding of uncertainty is informed by science and technology studies (STS). In this section, we provide a brief overview of how uncertainty is understood in this field. Our goal here is not to provide an exhaustive overview of sources of uncertainty, nor to provide an ironclad definition of the concept. Uncertainty can have different meanings in different contexts, and it is useful to keep an open view on the concept when describing how regulators deal with uncertainties in their decision-making practices (5).

Within STS, scientific knowledge is seen as inherently uncertain, because scientists never have direct access to the phenomena they are studying. The knowledge claims that scientists make based on their experiments can be more or less closely linked to their experimental setup, but these claims are always based on multiple steps of inference (15). For instance, suppose that one claims that a drug can treat diabetes based on a clinical trial that showed a reduction of HbA1c levels (which measures average blood sugar levels in the past few months) in the treatment group compared to placebo. This claim relies on a number of assumptions, including that the experimental procedure was adequate to measure HbA1c levels and that the observed difference between treatment arms can be attributed to the drug and not to another cause. Moreover, the claim of the drug’s effect relies on the assumption that a reduction of HbA1c levels is relevant in the treatment of diabetes.

Because the validity of the inferential steps that a claim relies on can be questioned, it is always possible to challenge scientific claims and to find alternative explanations for an experimental result. Collins refers to this phenomenon as experimenter’s regress. The assessment of the quality of an experiment depends on its results. A good experiment is one that detects a phenomenon, if it exists. However, if the phenomenon does not exist, a good experiment is one that returns a negative result. In other words, the experiment is designed to test the truthfulness of the claim, but it is only possible to assess whether the experiment was valid if the truthfulness of the claim is already known. The competence of the experimenter or the adequacy of the experimental procedure can therefore always be questioned when the results of an experiment are not in line with the orthodox scientific view (16).

Although it is always possible to challenge scientific claims, uncertainties that were once present in the knowledge production process become increasingly difficult to access once more knowledge is built on previous claims (17). Hence, it requires effort to reveal (or hide) uncertainties in knowledge claims. Uncertainties are not just “out there” (18).

In this paper, we explore the role of uncertainty in regulatory decision making in detail. Our analysis of the practice of uncertainty identification and evaluation is based on a larger ethnographic study of decision making at the Dutch Medicines Evaluation Board (MEB), see (19). Uncertainty became an important theme within this larger study.

## 2 Methods

### 2.1 Fieldwork

In our study, we mainly focused on the work of clinical assessors, who are responsible for assessing a drug’s efficacy and safety. At the MEB, the clinical assessors work in four different pharmacotherapeutic groups (PT-groups), organized per group of medical conditions. Group 1 assesses medicines in the areas focused on the central nervous system, eyes, anesthetics, the musculoskeletal system, analgesics, teeth, and skin. Group 2 focusses on cardiovascular diseases, diabetes, gynecology and hematology. Group 3 mainly focusses on oncology, and group 4 on vaccines, infectious diseases, gastrointestinal disorders, urology, and endocrinology (20). We followed the work of one of these groups (specific group not mentioned to maintain confidentiality).

Over the course of 11 consecutive months between 2019 and 2024 (precise dates not provided to reduce identification risks), the first author (JH) “shadowed” two drugs through different assessment rounds. To maintain confidentiality, we do not provide the actual (trade or generic) name for these drugs, but refer to them by the pseudonyms Zeranyl and Corzumide. These cases were chosen in close collaboration with two members of one of the MEB’s PT-groups, mostly for practical reasons. The assessment of Zeranyl was a decentralized procedure with a well-established use legal basis [article 10a procedure, see (1)]. This means that the drug was already used widely for a specific indication in clinical practice in at least one EU country for at least 10 years and that the drug’s dossier contained published scientific literature instead of new clinical data. The assessment of Corzumide was a centralized accelerated procedure [Article 8 (3) procedure] for which the Netherlands was one of the rapporteurs. JH attended meetings where these drugs were discussed. The observations took place online, because the regulators at the MEB primarily worked remotely at the time of study. JH also asked whether the clinical assessors could keep her up to date about any developments (e.g., by cc-ing her on relevant emails). Moreover, she had access to the written reports regarding the drugs and changes that were made to these reports over time.

In addition to focusing on these two drugs, JH attended the weekly meetings of the clinical assessor’s PT-group (where issues with any drugs currently under assessment by any assessor in the group were discussed); and she attended Board meetings (where the reports of all drugs currently undergoing assessment were discussed and approved). Through these observations, we therefore collected detailed fieldnotes about a large number of additional assessment cases beyond just the two drugs that were initially chosen to be shadowed, resulting in detailed notes of about 40 drugs. Another drug that we coincidentally followed through several assessment rounds, and discuss in some detail in this article, is Noradiol: a centralized indication extension procedure (type II variation) with the Netherlands as one of the rapporteurs.

In addition to observing meetings, JH conducted eight interviews with Zeranyl’s and Corzumide’s clinical assessors, a methodological assessor, a CHMP member and two Board members. The interviews were conducted via video meeting or phone and lasted 50–90 min. The interview template was amended with questions relevant for the specific person before each interview. Table 1 provides an overview of the material our analysis is based on.

**Table 1:** Overview of material.

Type of meeting	Number of meetings	Duration of each meeting
Board meeting (large)	5	5–6.5 h
Board meeting (small)	3	2.5–3.5 h
PT-meeting	41	1 h
Other meetings/phone calls	12	1–2 h
Semi-structured interviews^1^	Four clinical assessors	50–90 min
	One methodological assessor	
	Two Board members	
	One CHMP member	

^1^One of the eight interviewees had had multiple roles at the MEB. We only included their current role to reduce identification risk.

Both during Board meetings and PT-meetings, most of the meeting time was allotted to discussion of cases that regulators had questions or doubts about. As such, our analysis is largely based on material from more difficult assessment cases. According to one of our interviewees, about one in four cases takes a lot of work, while the other cases are more straightforward. The discussions we present in this article should therefore not be seen as representative for all evaluations conducted by the MEB. However, focusing on cases of doubt or disagreement is informative because these cases can reveal through which processes knowledge claims become accepted or questioned. In more straightforward cases, such processes remain hidden (21). Thus, the regulators’ discussions about difficult assessment cases can show what makes regulators have doubts about the validity, strength, or interpretation of the presented evidence (e.g., what makes regulators inclined to identifying uncertainties).

#### 2.1.1 Board meetings

The MEB’s Board consists of a maximum of 17 people who fulfill the role of Board member next to their work as a professional in the medical or pharmaceutical field. The Board convenes every 2 weeks to hold either a large Board meeting, where all Board members are present, or a smaller one that only a few members attend. In this smaller meeting, the few Board members that are present represent the rest of the Board. Similar issues are discussed during both types of Board meetings, although first round assessments where the Netherlands is (co-)rapporteur are preferably discussed during the large Board meeting. Assessment reports that will be discussed during the Board meeting are distributed prior to the meeting, together with a supplementary note that shortly summarizes the assessment, contains an overview of the most important OCs and MOs, and indicates the most pressing issues that the Board should discuss.

During the Board meetings, about 10–15 min was allotted to the discussion of each case. Each case was introduced by a project leader who shortly summarized the assessment and introduced the most pressing issue. The Chair of the Board then asked the Board members whether they agreed with the assessors on each discussion point brought up by the project leader. Board members could offer their opinion on the case and ask questions of the assessment team. The Dutch CHMP representatives and the assessment team were also present during the Board meeting and could offer their opinion on the case as well. Sometimes none of the regulators had a comment and the Chair quickly moved on to the next discussion point. At other times, an issue initiated a discussion between regulators. At the end of the discussion of a case, the Chair typically shortly summarized, based on the discussion, whether amendments should be made to the assessment or to the list of questions.

#### 2.1.2 Pharmacotherapeutic group meetings

During the weekly PT-meetings, a group of about 15 clinical assessors discussed the cases they were working on. Typically, assessments were carried out by one person, although sometimes multiple assessors worked on a case (as happened for both Zeranyl and Corzumide), depending on the size or complexity of the case. During each meeting, the chair of the meeting went through the different lists of drug assessments that members of the group were currently working on or that needed to be divided among the assessors. They paid specific attention to cases that would be discussed at the next Board meetings. The assessors involved in these cases would give a brief overview of their assessment and sometimes ask questions of the other participants of the meeting. Sometimes a discussion about difficulties they encountered during the assessment would follow.

#### 2.1.3 Ethics

For reasons of confidentiality, we use pseudonyms to refer to drugs and to regulators. Sometimes, we mention the specific role a regulator has (e.g., Board member or clinical assessor). However, some functions at the MEB and EMA are carried out by a limited number of people (e.g., CHMP representative). Mentioning their role would make them identifiable, so we only refer to them as “regulators.” Table 2 provides an overview of pseudonyms we use in this article and these regulators’ role at the MEB. Our article contains excerpts from fieldnotes, which are paraphrased; and it contains direct quotes from regulators. After each excerpt, we have indicated its document number of our Atlas.ti file.

**Table 2:** People who are mentioned multiple times in our article.

Role	Pseudonym
Clinical assessors working on Zeranyl	Jessica, Kees, and Stan
Clinical assessors working on Corzumide	Chris, Yvonne, and Petra
Other members of the PT-group	Lisette, Irene, Pepijn, Casper, Nadya, and Amanda
Board members	Gijs, Elias, Maarten, Sander, and Coen

Before the start of this study, MEB’s Board, the management of the MEB’s Agency, and the MEB’s scientific committee approved our research proposal. In addition to approval by the MEB, the study was approved by the Ethical Committee of the Department of Psychology at the University of Groningen (PSY-1920-S-0378). This article was checked for inaccuracies and potentially sensitive information following an internal peer-reviewing process of the MEB. Moreover, over the course of our study, we had several meetings with a small group of MEB regulators to discuss our results. We have incorporated the MEB’s feedback in our analysis.

#### 2.1.4 Analysis

Our analysis procedure is best captured as “reflexive thematic analysis,” see (22, 23). Braun and Clarke (23) emphasize the importance of flexibility, creativity, reflection, and researcher subjectivity in the process of generating themes, which they define as “patterns of shared meaning underpinned by a central organizing concept” (p. 593). Here we provide a brief description of our iterative process of coding and analysis, which was guided by the research questions of our larger ethnographic study and, later, also by the concept of uncertainty described in our introduction.

The aim of the larger ethnographic study was to describe how the clinical benefit–risk decision is made in practice at the MEB, and to describe what influences the regulator’s decision making. We specifically focused on what regulators valued in their decision-making practice. The idea that uncertainty played an important role in the regulator’s assessment of evidence was developed early on during JH’s fieldwork at the MEB. To illustrate, after about 2 months of observing meetings, she wrote down: “Doubts and discussions often revolve around uncertainties—things the studies cannot provide a conclusive answer to.” After another 2 months, JH started coding her material when she was still observing meetings at the MEB. The initial coding process was mostly inductive, meaning that codes were derived from the material. Informed by the initial coding of part of the material, but also through general impressions of observations and through discussions of the material with the co-authors, JH came up with eight main topics to describe the practice of drug assessment at the MEB that she later merged into three main themes related to uncertainty, consistency, and a drug’s value for clinical practice. These three main themes were then used to code the material three more times (each time focusing on another theme). While coding, JH started writing outlines to write up the “story” of each theme. This process was again recursive: the outlines were based on our theoretical assumptions, on the coding scheme, and on JH’s overall understanding of the material; and the coding was informed by the writing of these outlines. One of the three themes we developed forms the basis of this article. All three themes are discussed in detail in JH’s dissertation, see (19).

## 3 Results

Our qualitative analysis highlights the work-intensive and context-dependent nature of uncovering uncertainties. In the following, we first illustrate that regulators at the MEB use a “detective approach” to evaluate the claims an applicant makes about a drug in the submitted dossier. Evaluating drugs entails checking the applicant’s account of the evidence and actively searching for uncertainties that may weaken the claims about the drug’s effects. Thus, consistent with prior work (18), uncertainties are not just “out there,” but they require effort to uncover. We then discuss what makes regulators inclined to identifying uncertainties when they are scrutinizing a drug’s dossier.

### 3.1 Investigative approach: looking for uncertainties

Regulatory authorities typically do not collect data themselves. Instead, they work with a dossier of evidence composed by the applicant. The documents submitted by the applicant do not just contain data; they also give an interpretation of the data. Unlike the American Food and Drug Administration (FDA), the EMA does not routinely re-analyze raw data files. Regulators at EMA (and at the MEB) thus need to rely on the data collected, analyzed and presented by the applicant [although the applicant needs to adhere to strict requirements, see (24)].

An applicant wants the regulator to approve the drug, which can have implications for the applicant’s choices in the design of a study and in the presentation of its results. Regulators at the MEB are keenly aware that the dossier that is presented to them is not neutral. For instance, regulators mentioned that the dossier submitted by an applicant was written “toward a positive assessment” (fieldnote, Board member, D54). The applicant’s report is not seen as completely objective: “the overview written by the applicant provides a colored view” (quote, clinical assessor, D54). Thus, the regulator needs to see through the narrative presented by the applicant: “But the applicant’s style, we can see through that, right” (quote, regulator D54)?

It is the regulator’s task to scrutinize the information provided to them to verify whether the applicant’s claims are substantiated by the underlying evidence: “The company provides the results and then you go and check whether, indeed, they are accurate” (quote, Yvonne, D33). Clinical assessors check the dossier in detail because “you do not want to overlook anything” (quote, Petra, D21). This explains why one regulator talked about their job as “detective work,” which involves “digging” through the data (quote, Pepijn, D67). When assessing the medicine’s dossier, the assessors actively look for things in the dossier that are “remarkable,” “odd” (quotes, Yvonne, D8), “strange” (quote, Pepijn, D67) or “weird” (quote, Petra, D8). Are there maybe “irregularities that are not quite proper” (fieldnote, clinical assessor, D6)? In contrast, the presentation of the information by the applicant can be “tidy” (quote, Pepijn, D67) or good: “they have described everything very well, in great detail. (…) Everything looks very comprehensive” (quote, Yvonne, D8). Thus, regulators are checking the credibility of the claims that are being presented in the dossier.

In addition to assessing the credibility of the dossier’s claims, regulators scrutinize a dossier to select information they deem relevant for their benefit–risk assessment. They decide what information they find important to make credible claims about the drug. Regulators have certain questions in mind that they want to see answered by a drug dossier. When assessing the drug dossier, they need to decide which pieces of information answer their questions. They filter out what is relevant to them. They need to think about: “what question would you like to have answered? And what type of analysis is appropriate for that” (quote, Pepijn, D67)? The information regulators find important may not correspond to the information the applicant has chosen to emphasize in their narrative about the drug’s favorable and unfavorable effects. Regulators thus need to check whether the “correct data” (fieldnote, clinical assessor, D54) was used to come to the interpretation presented by the applicant. The assessors may for instance take another combination of endpoints into account in their judgment of the benefit–risk balance than suggested by the applicant; or the assessor of the case may deem a graph or table that is “hidden” in the report more important for the assessment than the prominently presented data.

Thus, regulators at the MEB sift through the submitted dossier to scrutinize the design and presentation of studies and to filter out the information they deem relevant for their assessment. Through this investigative approach, regulators assess the credibility of the applicant’s claims, and they identify uncertainties that may decrease the confidence they have in these claims. The remainder of the results section focusses on the main questions this article aims to answer: what makes a claim convincing to regulators; and in contrast, what makes regulators inclined to questioning a claim? We describe how regulators identify and evaluate uncertainties in practice. We contend that it becomes more difficult to question the applicant’s claims (1) when effects are “clearly visible”; or (2) when the presented evidence conforms to the regulator’s expectations. The different aspects we will discuss that make claims more or less convincing to regulators are not mutually exclusive. Coherence between different pieces of evidence is important.

### 3.2 Clearly visible effects

When talking about a drug’s effects, regulators often used the Dutch verb “zien,” which translates to both the active English verb “to see” and the more passive “to look like” or “to appear”: “it looks positive” (fieldnote, Jessica, D53). Efficacy should be clearly seen whereas major safety issues should not be seen, although not seeing unfavorable effects does not mean that regulators automatically accept that the drug is safe. They also consider the possibility that studies were simply unable to detect unfavorable effects, for instance, due to a limited study duration. Regulators can “see” an effect based on multiple sources of information. They may for instance see an effect based on statistical significance, an absolute or relative difference, or comparison to a pre-determined value (all of which may be presented either in text, tables or graphs). What the regulators see can be more or less convincing: “for me it is also quite convincing what we see” (quote, Gijs, D50).

Claims about a drug’s effects seemed to be more convincing to regulators if these effects were large and thus clearly visible. Hence, although unexpectedly large effects can lead to uncertainty about the validity of the finding (because they contradict expectations, as we will discuss below), large effects generally reduce the importance of uncertainties for the benefit–risk assessment.

A large, or clearly visible, effect increases the scientific credibility of a claim because it is difficult for regulators to completely attribute the effect to other potential causes than the drug. Looking for alternative explanations becomes difficult: “You can look for shades of gray, but you cannot get around that efficacy (…) in that case the assessment is almost black and white” (quote, Sander, D66). In that case, the magnitude of the effects “leaves no room for doubting whether the effect can be attributed to the drug” (quote, Casper, D31). For example, during a study investigating the drug Corzumide, some patients underwent a medical procedure (not described in the protocol) that could have had an effect on the measurements used to determine the efficacy of the drug. Despite this protocol deviation, the regulators reasoned that the large effect could probably not be fully undone by these deviations:

It turned out that with some patients they measured a [surrogate marker used to determine the efficacy of the drug] after [a treatment unrelated to the drug]. Instead, they should have measured it before that treatment. And all of that can influence the outcome. In this case, it only concerned a few patients, and the effect is very clear, so overall (…) it will not matter much (quote, Yvonne, D33).

The assessment of the credibility of the applicant’s claims involves the search for uncertainties that could invalidate these claims. When effects are large, or clearly visible, it becomes more difficult for regulators to identify uncertainties that can provide an alternative explanation for the reported effect. On the other hand, when effects are small or not clearly visible, it is easier to attribute these effects fully to other causes. Therefore, uncertainties could easily shift the assessment from positive to negative or the other way around.

### 3.3 Conforming to the regulator’s expectations

We illustrated that large and clearly visible effects are difficult to completely attribute to alternative causes (e.g., to uncertainties). However, a large reported effect does not necessarily mean regulators are convinced about the drug’s benefits or risks. Another important aspect is alignment between the regulator’s expectations and the presented evidence.

Regulators have certain expectations about how drugs should work and how they should be studied. These expectations can be informed by previous experience, regulatory guidelines, scientific knowledge, and other evidence. When a regulator’s expectations do not align with what was done in the study or with the study results, this can lead to doubt about the presented claims. On the other hand, good alignment between the presented information and regulator expectations may decrease a regulator’s doubts about the observed effects. Expectations can therefore act as a lens through which regulators look at the dossier. In this section, we discuss how different types of expectations may affect regulators’ estimation of the credibility of an effect and point regulators toward the identification and evaluation of specific uncertainties.

#### 3.3.1 Presentation

First, the presentation of a drug’s dossier can align more or less with the regulator’s expectations. According to the regulators, an applicant can use several strategies to present their drug in a favorable way. The applicant could be selective: “they were quite selective in the presentation of the analyses” (quote, Pepijn, D67). Information that is less favorable could be “hidden away” (quote, Pepijn, D67; and quote, Yvonne, D8) by the applicant in less prominent places of the report, such as at the bottom of a page or in the back of the dossier. The applicant can also quickly gloss over findings that are less favorable by not describing them extensively, or by presenting this information in a bulk of other information: “hidden within all the information” (fieldnote, clinical assessor, D50). In addition, language can be used to paint a more favorable picture: “sometimes the company can use specific adverbs to paint a rosier picture” (quote, Yvonne, D33).

How the applicant analyzed and presented its data can therefore give rise to caution, as a methodological assessor explained: “the entirety was just messy, and that made us skeptical about all the other analyses we received” (quote, interview). Similarly, Yvonne explained how an applicant’s short answers made her scrutinize the dossier more:

Also, when they were answering the first-round questions (…) that wasn’t really satisfactory. The company kind of brushed it off quickly. (…) Then you get a bit of a gut feeling. And then you actually want to scrutinize everything even more (quote, D33).

Thus, it seems that the presentation of evidence can lead to a more or less investigative approach by assessors. This is illustrated by a discussion between assessors Petra and Yvonne about the heterogeneity of a specific effect of Corzumide in patient subgroups, which they observed in supplementary tables provided by the applicant. They thought it was strange that the applicant did not provide forest plots to show the heterogeneity in this effect, while they did show such plots for other effects, especially because the applicant did show this subgroup data during a pre-submission meeting with the regulators. Petra and Yvonne thought they understood why the applicant did not provide the forest plot they would have liked to see: “it does not look good on the forest plot, of course” (quote, Petra, D8). The following discussion took place while the assessors scrolled though the submitted files to try to find the information they needed:

Petra: In the report itself, in the body, they left out the information. (…) Yvonne: Quite important information. I think it is weird that they didn’t include it here as well. (…) Petra: No, that’s odd indeed. (Petra is searching in the documents, but she cannot find the information). No, it is not included in the results … that isn’t great. Yvonne: Why wouldn’t you just show it? Petra: Yes, very interesting (quote, conversation Petra/Yvonne, D8).

During a next meeting, Yvonne returned to this issue. She was wondering whether they should ask a question about the potentially smaller effect in a subgroup of patients:

I thought we should say something about that. But on the other hand (…) it is not lack of effect, because you still have a [certain percentage] decrease in this subgroup. (…) It is a bit strange, because they have visualized some subgroups in forest plots but not others (fieldnote, Yvonne, D12).

Petra added: “[specific data] is also completely hidden in a post-test table. Why? That is also a subgroup, right” (fieldnote, D12)? However, Chris wondered what the added value would be of asking for this information. He thought the effect was quite clear: “It seems to me that the effect is quite clear. That it drastically lowers your [levels], so I do not know what else you want to know” (fieldnote, D12) (also demonstrating the importance of clearly visible effects for evaluating uncertainties)? Yvonne explained that the hidden data created “just a gut feeling” (quote, Yvonne, D12). In this case, not presenting the data in line with the regulators’ expectations led to a feeling that something was wrong. Thus, how the evidence is presented in a dossier can point regulators toward uncertainties or increase uncertainties they already have.

#### 3.3.2 Execution of study

In addition to the presentation of the data, the execution of the study can also adhere to the regulator’s expectations to a greater or lesser degree. One recurring theme in the discussions JH observed was that the information regulators deemed relevant for the questions they had about the drugs did not (fully) match the information that was provided in the drug’s dossier (although it should be noted that we focused on less straightforward cases that are not representative of all cases).

When the study protocol does not match the expectations of the regulator, this can create uncertainty about what cannot be seen in the study report. As one clinical assessor put it: “If you do not take a temperature, you cannot find a fever” (quote, D15; original reference from The House of God by Samuel Shem). Does the drug not cause a (side) effect or did the applicant just not conduct the right measurements or analyses to reveal it? When studies do not adhere to the regulator’s expectations, regulators may explain effects that they (cannot) observe as being caused by an inadequate study design or execution.

This raises the question what expectations and wishes regulators have about a study’s design and execution. To assess the scientific credibility of an applicant’s claims about a drug, regulators are interested in getting a “clear picture” (quote, Petra, D21) of the drug, meaning that they are interested in a drug’s effects in isolation. Studies that provide regulators with a clear picture of the drug’s effect in isolation are studies that are randomized, that are blinded and that compare a drug to placebo; studies that did not use the drug in a combination-treatment; and studies that use a type of analysis that is less affected by alternative causes (e.g., an analysis that corrects for patients who discontinued treatment).

Moreover, to regulators, a convincing study design is a design that closely matches the situation in which the drug will be applied: “The most important data originate from those studies (…) that have actually examined the patients for which they propose the indication. (…) We will judge those kinds of data to be particularly suitable in such a dossier” (quote, Chris, D19). Thus, if the drug is for a first line treatment, regulators would preferably like to see a study conducted in the first line; if the drug should prevent certain clinical outcomes, the regulators would like to see data on clinical instead of surrogate endpoints; etc.

In addition, regulators expect studies to be executed in accordance with the plans that have been made beforehand. EMA’s scientific guidance “ICH E9 statistical principles for clinical trials” states that a clinical trial protocol should be clearly defined beforehand and that “The extent to which the procedures in the protocol are followed and the primary analysis is planned *a priori* will contribute to the degree of confidence in the final results and conclusions of the trial” (25, p.5, our emphasis in bold). Thus, failure to conduct a trial according to protocol can cause regulators to become doubtful about the presented claims.

Regulators can communicate their expectations about the appropriate study design to the applicant beforehand, for example through regulatory guidelines and scientific advice. When the submitted dossier does not adhere to these communicated expectations, this may again be a reason for doubt to arise. For instance, Yvonne noticed that the inclusion criterion for the trial of Corzumide was not in line with the current guideline and therefore not in line with the regulator’s expectations. Even though deviation from the guideline was understandable because the guideline was updated after the study had already started, Yvonne still found it strange because the chosen inclusion criterion (a certain level of a specific molecule) was not logical: “I could not completely reconcile that. It does not align with the mechanism of what this drug is supposed to do. (…) Normally, the inclusion criterion is based on [levels of another molecule]” (fieldnote, Yvonne, D8).

Thus, to assess the scientific credibility of a drug’s effects, regulators are interested in a study design that enables isolation of drug effects and that closely resembles the drug’s proposed indication. Such studies are of course not always achievable in practice (e.g., due to ethical reasons). Moreover, the applicant determines what studies to conduct, not the regulator (although the regulator may provide advice, and drug developers need ethics approval to run a clinical trial in the EU). Regulators are thus not always presented with the kind of evidence they would have liked to see to answer the questions they have about a drug. If studies do not adhere to the regulators’ expectations, this may lead to increased scrutiny by the regulator, even if the studies do adhere to pre-defined plans. The regulators’ expectations about study design can be driven by a combination of what the guidelines prescribe, by what is standard practice for the type of disease, and by what seems logical. In addition, regulator expectations about study design and results can also be influenced by previous experiences with the assessment of similar drugs, which is discussed next.

#### 3.3.3 Alignment with previous experiences

Depending on how long assessors have worked at the medicine regulator, they will have more or less experience with the review of drugs, which may shape how they look at new cases. Regulators may have assessed a similar case in the past, or they may have personal or professional experience with a drug, for instance with prescribing the drug to patients. A drug under review may be part of a larger drug family of substances that work in a similar way or it may already be approved for other indications. If this is the case, regulators are already somewhat familiar with how the drug (should) work(s) and what its possible (side) effects could be. This can set the expectations that assessors have about a case.

Previous experience with a drug or similar drugs can inform the regulator’s assessment of the credibility of the dossier’s claims. Uncertainty about the effects of a drug may decrease when previous experience aligns with the study results, making the assessment more straightforward: “The case is not very spectacular, essentially more of what we already knew. Yes, it works” (fieldnote, Kees, D34). This could make it less likely that results will be questioned. On the other hand, previous experience may increase uncertainty when it does not align with what has been presented to the regulators in the current case. In such a case, experience can point regulators to potential uncertainties that may partially invalidate the dossier’s claims.

For instance, the assessors of Zeranyl questioned whether the outcome of a trial could have been a chance finding because they compared its unexpectedly large effect size to what they had seen for other drugs to treat the disease:

Especially when you look at other drugs that have been registered for [this disease] in recent years. For example, when you look at [names of other drugs]. Those have studies with [a large number of] people, or much larger, and with a smaller effect than what has been measured now (fieldnote, Jessica, D57).

In this case, the large effect contradicted the regulator’s expectations based on previous, similar drugs and therefore led to caution. The clinical assessors wondered whether the outcome could have been a chance finding: “In a small study you sometimes find something that is just coincidental” (fieldnote, Stan, D55).

Because they had doubts about the validity of the effect, the regulators searched for explanations for the reported outcome. However, as explained before, it is difficult to attribute a large effect to alternative causes, which became clearly visible during the assessment of Zeranyl. During the second Board meeting where Zeranyl was discussed, several Board members (Sander, Elias, and Coen) were critical of the assessment report written by clinical assessor Jessica, which they thought was too positive: “I feel like there are more critical aspects we could consider related to that study. Perhaps we should formulate it more critically (…) I feel like there are all kinds of shortcomings to this study” (fieldnote, Sander, D60). Sander was, among other things, critical of the premature termination of the study and of the chosen primary endpoint. However, Kees explained that it was difficult to find arguments that could fully invalidate the study results: “I share your (Sander, Elias and Coen’s) concerns. However, thoroughly challenging this study is quite difficult. They do reach a hazard ratio of [#], which is quite impressive. Even if the number of patients in the study is [small]” (fieldnote, Kees, D60). Another Board member added:

If this is the only proof that the study is flawed, then you need to try very hard to completely explain away that hazard ratio. You would need a combination of arguments, including the argument just mentioned by Coen that the mechanism of action is not plausible. (…) The question is whether you can erase the entire effect with that (fieldnote, Maarten, D60).

Hence, even though the unexpectedness of the large effect caused regulators to feel doubt, they found it difficult to “explain away” such a large effect. This case illustrates how regulators may start looking for uncertainties when they have doubts about a case due to misalignment with their expectations. In addition, it illustrates the point we made at the beginning of the results section: different aspects that determine how convincing an effect is to regulators cannot be seen separately from each other. Coherence is important.

In addition to influencing the identification of uncertainties, regulators’ expectations about the drug, based on previous experience, can influence how regulators evaluate the importance of uncertainties. A discussion about the drug Noradiol illustrates this. Studies of the drug Noradiol showed an increase in serious side effects compared to placebo. However, the reported numerical difference in prevalence of this rare side effect was small. Whether or not the risk associated with Noradiol was actually increased was therefore questioned. During the first evaluation round, the Dutch regulators were positive about the benefit–risk ratio of the drug. However, the other rapporteur was negative and proposed an MO related to the increased risk. Chris used his previous experience assessing Noradiol for a different indication to motivate his reasoning for not putting too much weight on the increase in serious side effects: “Of course, such a study does not exist in isolation. (…) we know the safety issues of this drug” (fieldnote, Chris, D27). According to him, other studies of Noradiol did not show a difference in the prevalence of serious side effects: “there, we saw lower mortality rates and also slightly less of the [serious side effect]. And that was the same dosage. So if we start complaining now about a slightly different indication, but otherwise identical case, I would find that strange” (fieldnote, Chris, D32). Due to his previous experience, Chris expressed doubt about whether the reported imbalance in unfavorable effects could be attributed to the drug. It could be that people with a slightly more serious presentation of the disease were included in the treatment group: “there are only a few occurrences. A slight imbalance can already cause you to see this” (fieldnote, Chris, D32). Thus, based on previous experience, Chris seemed to evaluate the drug’s potential risk differently than the other rapporteur of the case who wanted to pose an MO about it. He advised his colleagues to steer the assessment toward the downgrading of the proposed MO: “You need to steer the [assessor of other country] a bit. Otherwise, this might head in the wrong direction, so direct it toward not keeping it as a MO” (fieldnote, Chris, D27).

Apart from regulatory experience, regulators can also have personal and professional experience with the drugs they review (e.g., with prescribing the drug to patients). Most Board members, and many assessors, have previously worked with patients or still work with patients. A misalignment between the claims in the study and how colleagues in the hospital think about the drug, or use the drug with patients, may therefore be another reason to question the dossier’s claims. For instance, both Zeranyl and Noradiol were already available to doctors and could therefore be used off-label for the new indications. However, doctors did not include Zeranyl in professional treatment guidelines. These guidelines were updated after the results of the pivotal study (the main study supporting the claims of the applicant in the dossier) were published, which made Jessica wonder:

One of the questions is also (…) how can it be explained that not everyone, every clinical practice guideline, is adopting this as their first choice based on these findings? Do you understand what I mean? There is a discrepancy somewhere between the findings of that study and the daily implementation in clinical practice (quote, Jessica, D16).

Similarly, after the results of the study for the new indication became known, a regulator explained that Noradiol “had not been welcomed with great enthusiasm by doctors” (fieldnote, D50). When other regulators asked assessor Irene how Noradiol was viewed in practice, she answered that doctors will most likely not use it for the new indication. According to her: “[the registered alternative drug] continues to be the preferred choice of doctors” (fieldnote, Irene, D50). Whether or not a drug is used in clinical practice does not determine the regulator’s decision, but, as these examples show, it can make regulators question a drug’s claimed effects.

#### 3.3.4 Alignment with scientific knowledge

Expectations about plausible effects can be based on previous experience regulators have with a (similar) drug, but they can also result from the scientific knowledge base of theories and experimental evidence describing how molecules in the body interact and how the drug potentially influences this interaction to prevent disease symptoms or cause side effects.

Reasoning based on the mechanism of action explains how the drug potentially causes the claimed effects and embeds study results into a larger framework of biological explanations (26, 27). Effects are more convincing if regulators believe in the mechanism by which the drug is supposed to work, as Sander explained: “Of course it encompasses more than just some clinical data. Whether you believe in the underlying mechanism is also important” (quote, Sander, D66). Understanding the mechanism can increase confidence in the effects when this aligns with what the data shows; alternatively, it may decrease confidence when there is an inconsistency.

Similar to the mechanism of action, a discrepancy between other published data and the applicant’s data can increase or decrease uncertainty. For example, Yvonne explained that an applicant claimed that a subgroup of patients with low biomarker levels would benefit most from the drug she was reviewing. However, she also found scientific literature claiming that this group of patients actually has an increased risk of mortality: “so there is friction, that does not completely add up. I could not fully reconcile that” (quote, Yvonne, D34).

Scientific knowledge can also help regulators to evaluate the importance of potential uncertainties. When the rationale for how the drug causes its effects is clear, identified uncertainty about the reported effects may be less important for the decision. For instance, when talking about a drug that was conditionally approved without data from a randomized controlled trial (RCT), Gijs explained: “there was also a logical rationale supporting why it would work” (fieldnote, D60). In this case, the uncertainty caused by the lack of data from RCTs was compensated by a logical rationale through which the drug should work. Similarly, knowing what a drug does in the body can fill in some gaps in knowledge that the drug dossier cannot resolve. For instance, when discussing whether half the dosage of a drug for a liver disease would be just as effective in children as a full dosage in adults, a Board member reasoned: “if the clearance is higher in children, and I think that is the case, then the exposure in the liver is even higher, so in that case you do not have to worry about the efficacy at all” (fieldnote, Coen, D35).

Scientific knowledge can thus help to identify and evaluate uncertainties. However, when the scientific knowledge itself is incomplete, contradictory, or lacking, this may also increase uncertainty about the drug’s claimed effects. This can for instance happen when the data shows that a surrogate endpoint (e.g., blood levels) is lowered, but scientific knowledge about a clinical endpoint (e.g., the relationship between lowering these blood levels and patient symptoms or disease progression) is lacking. Both mechanistic and clinical data could help to strengthen this link, but if this information is lacking, uncertainty can arise about the clinical meaningfulness of the drug’s effects: “The problem, at least for me, is that you do not know whether the elevated [levels] have any impact on organ improvement” (fieldnote, Nadya, D40).

#### 3.3.5 Alignment between pieces of evidence

Finally, misalignment between different pieces of information in the drug’s dossier can open the door to alternative explanations. There can be variations in the results of multiple clinical trials included in the dossier, or differences between clinical and non-clinical studies. Moreover, results within a single study can be heterogenous, for instance for different doses or subpopulations included in the study. Furthermore, the results of a study on various endpoints could be more or less convincing or even may point in different directions.

Because variations in results are not expected, they need to be explained. For instance, the clinical assessors of a drug noticed that there was a difference between the levels of [substance A] and the levels of [substance B] reported in the trial. While the levels of [A] went down and remained low, a graph showed that the levels of [B] initially went down but after a while increased toward levels higher than seen in the placebo group:

Amanda: there is a sort of mismatch between [A] and [B]. The [levels of substance A] improve, but [B] returns to the initial level. That is what you cannot explain?

Nadya: no, I don’t have an explanation for that (fieldnote, D42).

Kees tried to explain the difference by reasoning with reference to the mechanism of action. He explained what substance B does in the body and how it is renewed over time. Another regulator wondered whether the experimental protocol could explain the outcome: “because if I look at that data, it seems like they simply discontinued the medication after [number of] weeks” (fieldnote, D42). To gain clarity, the assessors asked the applicant to explain what could have caused these seemingly contradictory results: “just let the company explain that” (fieldnote, Lisette, D42).

Misalignment between different pieces of evidence raises questions for the regulators, such as: are the results from these studies comparable or can the effect be explained by differences in study design (heterogeneity in effect or in study design)? Is the same (chemically equivalent) drug used in each study? Is there another logical reason that could explain differences in effects between studies or subgroups? Variation of evidence can be explained by the complete absence of an effect, by partial absence of an effect (e.g., the drug only works in certain subpopulations), by the nature of an effect (e.g., how clearly it can be measured), or by a variety of methodological issues.

### 3.4 Individual differences

The previous sections have described what makes a claim convincing to a regulator or what makes them more inclined to questioning a claim. We also illustrated how regulators use their knowledge about previous assessments or the scientific literature to assess the credibility of the claims presented in the drug’s dossier. It therefore makes sense that different regulators may disagree on whether a claim is convincing or not; what is uncertain about a claim; and how important these uncertainties are.

Individuals may evaluate the scientific credibility of claims based on the same evidence differently. Some regulators may see an effect whereas others are not convinced that an effect exists. In addition, regulators may see the uncertain nature of the evidence differently. As this article illustrates, evidence and uncertainties are interrelated. Whether someone is convinced that there is an effect also depends on what uncertainties they have identified and how they interpret these. There may be disagreement about the interpretation of effects and uncertainties in relation to each other:

We state that there is a small effect, but I am not yet convinced that there is an effect. The outcome measure is scale dependent (it matters whether you look at absolute or relative numbers). They use relative reduction here, but if you measure the absolute reduction, there is no difference with placebo. Furthermore, with the percentages (…) the outcome is very dependent on the people they no longer include. (…) I am not yet convinced that there is an effect (fieldnote, Maarten, D49).

In this example, Maarten was not convinced of the existence of an effect, since the reported outcome can also be explained by the analysis procedure. Thus, one person may be convinced that there is an effect, whereas another person may be convinced that the reported effect can be explained by other causes.

In addition, even if regulators agree about specific uncertainties present in the applicant’s claims, they may still disagree about the importance of these uncertainties. This can be seen in discussions about whether an uncertainty should be formulated as an OC or as an MO. Because an MO can potentially block approval when it is not resolved, posing an MO gives more importance to the uncertainty than an OC would. Discussions about the “up-or downgrading” (fieldnote, multiple Board meetings) of MOs or OCs show how regulators try to reach consensus on the importance of uncertainties. For example, when discussing a case where the Netherlands was not one of the rapporteurs, the regulators explained that one of the rapporteurs wanted to pose an MO regarding protocol deviations. However, the Dutch assessors did not agree: “The Netherlands judges it more as an OC, since it will not significantly change the results of the study” (fieldnote, D50). They thus wanted to downgrade this MO to an OC. While regulators may agree that something in the dossier is uncertain, they may disagree on how uncertain it is and on how important this uncertainty is.

## 4 Discussion

In this article, we have aimed to shed some light on the “regulatory thought process” (28, p.144) by describing how regulators at the Dutch MEB evaluate the credibility of a drug’s reported effects in practice.

We have shown that regulators scrutinize the applicant’s account of the evidence and are actively searching for uncertainties that may weaken the claims about the drug’s effects. In line with Moreira (21) and Van Loon and Bal (29), our account illustrates that it requires effort to uncover uncertainties. In addition to showing the effort it takes to uncover uncertainties, we have described under what circumstances the uncovering of uncertainties takes more or less effort. In other words: what makes a claim convincing to regulators, and in contrast, what makes them inclined to question a claim?

We argue that it becomes more difficult to question the applicant’s claims (1) when effects are “clearly visible”; or (2) when the presented evidence conforms to the regulator’s expectations. Regulators’ expectations about the presentation, execution and results of studies can shape how they look at a medicine’s dossier. Questioning the reported effects of a drug takes more effort when a dossier is tidy and coherent; when it contains studies that have been conducted according to the regulators’ expectations; or when it contains consistent information or results that align with the regulator’s experience or scientific knowledge.

It is important to note that the various aspects that may influence the regulator’s evaluation of the credibility of an applicant’s claims should not be seen in isolation. When an effect is small or not clearly visible, it is easier to attribute it completely to alternative causes. In those instances, alignment between different pieces of evidence and the regulator’s expectations may become more important for the evaluation. Similarly, when the presented evidence does not conform to the regulator’s expectations in one specific regard (e.g., the dossier looks messy); it may make them more inclined to scrutiny of the entire dossier.

Our study sheds light on the context-dependent nature of uncertainty handling (4). We have shown how regulators use their knowledge about previous assessments or the scientific literature to assess the credibility of the claims presented in the drug’s dossier. As Van Asselt (5) explains, previous experiences may help people make sense of uncertainties by pointing toward the likely ways in which the uncertainties will play out or toward the uniqueness of the situation under deliberation. This means that individuals may interpret the credibility of presented claims differently, as we have illustrated. Thus, in addition to variation in the identification of uncertainties between different regulatory organizations (e.g., see 30); individuals within one organization may identify different uncertainties.

While our article provides more insight in how regulators assess the credibility of a drug’s claims and identify uncertainties, we did not focus on how regulators subsequently deal with uncertainties. However, during our ethnographic study, we did observe multiple strategies regulators could resort to. During the assessment procedure, regulators may ask the applicant for additional data, analysis, or justification to gain more clarity about uncertainties. When uncertainties cannot be resolved, regulators may request post-approval data collection. Moreover, regulators may try to reduce the scope of an applicant’s claims by aligning a drug’s indication with the study’s included population, meaning that those claims are based on fewer inferential steps, and thereby become more credible (e.g., see 15). In addition, regulators may propose strategies to handle the risk that is associated with uncertainties. Uncertainty carries risk when it is unclear whether a particular situation may occur, but it is clear that some of the potential situations are undesirable (5). For example, to handle uncertainties, regulators may advise that patients who take the drug are closely monitored so that the treatment can be stopped or adjusted when safety or effectiveness is disappointing.

Additional uncertainty mitigation strategies that we did not observe, but that may be useful for regulators, could focus on trying to avoid uncertainty. For instance, Hogervorst et al. (4) have developed a tool that stakeholders, such as regulatory authorities and drug developers, could use to anticipate potential uncertainties and to identify appropriate strategies to prevent or mitigate these. Based on our analysis we provide two more measures regulators could take to reduce avoidable uncertainty.

First, regulators could decide to rely less on evidence produced by the drug developer. As we have illustrated, regulators at the MEB seemed to approach the applicant’s claims with caution. This approach closely resembled the description by Brown et al. (31) of the default state of skepticism exhibited by health technology assessment (HTA) committee members toward the applicant’s presented information. Although HTA organizations focus on a benefit–cost assessment instead of a benefit–risk assessment, they also rely on information presented to them by an applicant, which Brown et al. observed gave rise to uncertainty. Some uncertainty could therefore be avoided by relying less on studies designed, and presented by the applicant or on their behalf [e.g., by contract research organizations who can be hired by companies to conduct clinical trials for them, see (32)]. This suggestion is not new. For example, Garattini and Bertele’ (33) propose that regulators should require each drug dossier to contain at least one phase III trial designed and carried out by an independent, and regulator approved, organization. The difficulty with this proposal is how to fund these trials while making sure that choices of the design and analysis of these studies are made independently from the funder. In addition to requiring independently conducted trials, regulators could collect and analyze data themselves. An example of this is the initiative DARWIN EU, which collects real world observational data that the EMA can use to set up non-interventional studies to support their decision-making (34), although this is of course not applicable to new drugs. Finally, the regulator could re-analyze the raw data the dossier is based on, which would allow them to check the quality of the data and to perform analyses relevant for the questions they have. The FDA already re-analyses data, so when the applicant applies for marketing authorization at both agencies, EMA regulators can to some extent rely on the FDA’s analysis as an assurance of the quality of the applicant’s performed analysis (35). A drawback of this approach, apart from the time and costs involved, is that the regulator would still rely on data produced by (or on behalf of) the drug developer.

A second way regulators could reduce avoidable uncertainty is through improved communication about, and enforcement of, desired evidence standards. A recurring theme we observed was that the executed studies did not (fully) adhere to the regulator’s expectations, leading to uncertainty about what the regulators could not see in the dossier (although, it should again be noted that our observations mostly concerned less straightforward cases). Regulators can indirectly try to influence what kind of studies drug developers execute, for instance through communication in guidelines and through scientific advice. During our observations, comments were made that point toward the potential improvement of this scientific advice, which, at the European level, is provided by the Scientific Advice Working Party (SAWP). During observations, assessors mentioned that the SAWP’s advice did not always align with their wishes. Clinical assessors expressed that the SAWP tends to be less strict in their requirements than they would like to be in their assessments. However, given its advisory role and its positioning *prior* to the start of the studies (when study design can still be altered), it would be more logical for the SAWP to be more, rather than less, demanding than the CHMP. The SAWP can express what studies the regulator would ideally like to see, while assessors will need to accept that these ideal studies may not have been possible in practice. As one regulator commented: “The SAWP’s advice takes place before registration, so you need to express what you consider ideal” (fieldnote, D37). Further investigation is needed to determine whether there truly is a difference between desired evidence standards that are communicated through scientific advice and the expectations of clinical assessors (also taking into account the expectations of regulators at NCAs other than the Dutch MEB). Such a study could then be used to improve alignment between the SAWP and CHMP.

Finally, we think it is important to note that uncertainty is not necessarily a bad thing. Uncertainty can be productive, as it can make people reconsider taken-for-granted assumptions. For example, Moreira et al. (18) show how in discussions by an FDA advisory committee, uncertainty allowed participants of the meeting to collaboratively reflect on existing conventions such as the definition of Alzheimer’s disease, which seemed partly arbitrary. Subsequently, the exposed uncertainties allowed for the reconstruction of new temporary conventions from which new research about mild cognitive impairment could be initiated. To make optimal use of the regulator’s investigative approach to question assumptions, and given the importance of experience and expertise in the regulator’s sensitivity toward specific uncertainties, it would be valuable for regulatory authorities to create assessment teams of people with diverse professional backgrounds.

### 4.1 Limitations

Our study comes with some limitations. As explained before, our analysis is largely based on material from more difficult assessment cases. Additionally, other considerations that are not related to the scientific credibility of the presented evidence may play a role in the regulator’s decision-making [such as consistency or a drug’s value to clinical practice, see (19)]. Therefore, the discussions we have presented in this article should not be seen as representative for all evaluations conducted by the MEB. Moreover, in our article, we have mainly focused on what makes a claim convincing to a regulator who is examining a drug’s dossier. However, medicine assessment at the MEB takes place in collaboration and discussion with others. It involves assessors, Board members, CHMP members, other European member states and the applicant. At the CHMP, decisions are often reached in consensus, meaning that individual differences need to be bridged (8). Moreover, the EMA and MEB use procedures (e.g., guidelines and templates) to try to ensure consistency between assessments and assessors. Future research should focus on increasing our understanding of how the different factors that potentially influence the identification and evaluation of knowledge claims eventually culminate into a single regulatory decision. To increase our understanding of regulatory decision making, it would be especially interesting to focus on the social processes involved in reaching consensus and to study decision making at different NCAs (and at the CHMP) that may organize their assessment procedures differently.

## 5 Conclusion

In this article, we highlighted the work-intensive and context-dependent nature of medicine evaluation. We showed that regulators at the MEB used an investigative approach to scrutinize a drug’s dossier. Thus, evaluating drugs entails checking the applicant’s account of the evidence and actively searching for uncertainties that may weaken the claims about the drug’s effects. In addition, we have described what makes regulators more or less inclined to identify uncertainties, in particular a large or clearly visible effect (which is difficult for regulators to completely attribute to alternative causes) and deviation from the regulator’s expectations about the presentation, design and results of a study. Our research has sought to increase our understanding of how context may influence a regulator’s judgment of a drug’s presented evidence and makes clear the importance of regulatory experience and expertise in the judgment of a claim’s credibility. Future research might focus on investigating the social process of medicine evaluation, which takes place in collaboration and discussion.

## Data availability statement

The datasets presented in this article are not readily available because the data contain identifiable information. Therefore, the data can only be accessed with permission of the authors and the Dutch MEB. Requests to access the datasets should be directed to j.m.hoek@uu.nl.

## Ethics statement

The studies involving humans were approved by Ethical Committee of the Department of Psychology at the University of Groningen (PSY-1920-S-0378). The studies were conducted in accordance with the local legislation and institutional requirements. Written informed consent for participation was not required from the participants or the participants’ legal guardians/next of kin because the manuscript was reviewed for sensitive/identifiable information by members of the MEB’s scientific committee prior to publication. Written informed consent was not obtained from the individual(s) for the publication of any potentially identifiable images or data included in this article because the manuscript was reviewed for sensitive/identifiable information by members of the MEB’s scientific committee prior to publication.

## Author contributions

JH: Conceptualization, Writing – original draft, Writing – review & editing, Data curation, Formal analysis, Investigation, Methodology, Validation. JB: Conceptualization, Supervision, Validation, Writing – review & editing. YV: Conceptualization, Supervision, Validation, Writing – review & editing. RM: Conceptualization, Supervision, Validation, Writing – review & editing. DR: Conceptualization, Funding acquisition, Project administration, Supervision, Validation, Writing – review & editing.
