# Erratum: Assessing the credibility of a drug's effects: identification and judgment of uncertainty by the Dutch Medicines Evaluation Board

**DOI:** 10.3389/fmed.2024.1486408

**Published:** 2024-09-19

**Authors:** Frontiers Production Office

**Affiliations:** Frontiers Media SA, Lausanne, Switzerland

**Keywords:** uncertainty, benefit–risk, regulatory decision making, credibility, regulatory authorities

Due to a production error, certain citations in the article did not refer to the correct reference.

Original reference 29. Shapin S. Cordelia's love: Credibility and the social studies of science. *Perspect Sci*. (1995) 3:255–75. has now been corrected to reference 15.

Original reference 15. Collins H. *Changing Order: Replication and Induction in Scientific Practice*. Chicago, IL: University of Chicago Press (1992). has now been corrected to reference 16

Original reference 16. Latour B, Woolgar S. *Laboratory Life: The Construction of Scientific Facts*. Princeton, NJ: Princeton University Press (1986). has now been corrected to reference 17.

Original reference 17 Moreira T, May C, Bond J. Regulatory objectivity in action: mild cognitive impairment and the collective production of uncertainty. *Soc Stud Sci*. (2009) 39:665–90. doi: 10.1177/0306312709103481 has now been corrected to reference 18.

Original reference 18. Hoek JM. *What is a good decision in medicine evaluation? Unravelling the regulatory though process* (Dissertation). Groningen: University of Groningen (2024). has now been corrected to reference 19.

Original reference 19 College ter Beoordeling van Geneesmiddelen (2019). *Pharmacotherapeutic groups—the agency-Medicines Evaluation Board*. College ter Beoordeling van Geneesmiddelen. Available at: https://english.cbg-meb.nl/topics/about-meb-the-agency/about-meb-pharmacotherapeutic-groups has now been corrected to reference 20.

Original reference 20 Moreira T. Health care rationing in an age of uncertainty: a conceptual model. *Soc Sci Med*. (2011) 72:1333–41. doi: 10.1016/j.socscimed.2011.02.026 has now been corrected to reference 21.

Original reference 21 Braun V, Clarke V. Using thematic analysis in psychology. *Qual Res Psychol*. (2006) 3:77–101. doi: 10.1191/1478088706qp063oa has now been corrected to reference 22.

Original reference 22 Braun V, Clarke V. Reflecting on reflexive thematic analysis. *Qual Res Sport Exerc Health*. (2019) 11:589–97. doi: 10.1080/2159676X.2019.1628806 has now been corrected to reference 23.

Original reference 23 European Council. *Regulation (EU) no 536/2014 of the European Parliament and of the council of 16 April 2014 on clinical trials on medicinal products for human use, and repealing directive 2001/20/EC*. Off J Eur Union 57(L158). (2014). Available at: https://eur-lex.europa.eu/eli/reg/2014/536/oj/eng has now been corrected to reference 24.

Original reference 35 European Medicines Agency. *ICH topic E 9-statistical principles for clinical trials-step 5*. London. Report No.: CPMP/ICH/363/96. (1998). Available at: https://www.ema.europa.eu/en/documents/scientific-guideline/ich-e-9-statistical-principles-clinical-trials-step-5_en.pdf has now been corrected to reference 25.

Original reference 24 Howick J, Glasziou P, Aronson JK. Problems with using mechanisms to solve the problem of extrapolation. *Theor Med Bioeth*. (2013) 34:275–91. doi: 10.1007/s11017-013-9266-0 has now been corrected to reference 26.

Original reference 25 Thompson RP. Causality, theories and medicine. *Causal Sci*. (2011):25–44. has now been corrected to reference 27.

Original reference 26 Tafuri G. (2013). *Exploring the regulatory decision-making process for medicines* (Dissertation). Utrecht: Utrecht University. has now been corrected to reference 28.

Original reference 27 van Loon E, Bal R. Uncertainty and the development of evidence-based guidelines. *Valuat Stud*. (2014) 2:43–64. doi: 10.3384/vs.2001-5992.142143 has now been corrected to reference 29

Original reference 28 Vreman RA, Naci H, Goettsch WG, Mantel-Teeuwisse AK, Schneeweiss SG, Leufkens HGM, et al. Decision making under uncertainty: comparing regulatory and health technology assessment reviews of medicines in the United States and Europe. *Clin Pharmacol Ther*. (2020) 108:350–7. doi: 10.1002/cpt.1835 has now been corrected to reference 30

Original reference 30 Brown P, Hashem F, Calnan M. Trust, regulatory processes and NICE decision-making: appraising cost-effectiveness models through appraising people and systems. *Soc Stud Sci*. (2016) 46:87–111. doi: 10.1177/0306312715609699 has now been corrected to reference 31

Original reference 31 Sismondo S. *Ghost-Managed Medicine: Big Pharma's Invisible Hands*. Manchester: Mattering Press (2018). Has now been corrected to reference 32

Original reference 32 Garattini S, Bertele V. How can we regulate medicines better? *BMJ*. (2007) 335:803. doi: 10.1136/bmj.39281.615706.94 has now been corrected to reference 33

Original reference 33 European Medicines Agency. *Big data steering group (BDSG): 2020 report*. Report no.: EMA/48625/2021. (2021). Available at: https://www.ema.europa.eu/en/documents/report/big-data-steering-group-bdsg-2020-report_en.pdf has now been corrected to reference 34

Original reference 34 Tafuri G, Stolk P, Trotta F, Putzeist M, Leufkens HG, Laing RO, et al. How do the EMA and FDA decide which anticancer drugs make it to the market? A comparative qualitative study on decision makers' views. *Ann Oncol*. (2014) 25:265–9. doi: 10.1093/annonc/mdt512 has now been corrected to reference 35

The publisher apologizes for this mistake.

The original article has been updated.

